# P-1648. Efficacy of Co-Ultramicronized Palmitoylethanolamide With Luteolin for Managing Post-COVID-19 Persistent Olfactory Dysfunction: A Systematic Review and Meta-Analysis of Randomized Clinical Trials

**DOI:** 10.1093/ofid/ofaf695.1823

**Published:** 2026-01-11

**Authors:** Omar Elkoumi, Ahmed Elkoumi, Mariam Khaled Elbairy

**Affiliations:** Faculty of Medicine, Suez University, Suez, Egypt, Suez, As Suways, Egypt; Faculty of Oral and Dental Medicine, Egyptian Russian University, Badr City, Al Qahirah, Egypt; Faculty of Medicine, Suez University, Suez, As Suways, Egypt

## Abstract

**Background:**

Persistent olfactory dysfunction (OD) after long COVID-19 is an emerging global health concern. However, evidence-based therapies for persistent OD post-COVID-19 remain limited. This meta-analysis aims to assess the effectiveness of co-ultramicronized palmitoylethanolamide with luteolin (um-PEA-LUT) combined with olfactory training (OT) for managing post-COVID-19 OD.

**Methods:**

PubMed, Scopus, Web of Science, Embase, and Cochrane databases were systematically searched. Randomized clinical trials (RCTs) comparing um-PEA-LUT plus OT versus OT alone in treating post-COVID-19 OD were included. The primary outcome was the change in Sniffin’ Sticks test scores for Threshold, Discrimination, and Identification (TDI). Statistical analysis was performed using Review Manager software, and the results were presented as the mean difference (MD) with 95% confidence intervals (CI), employing a random-effect model.

**Results:**

Four RCTs with a total of 367 patients were included in the meta-analysis. The combination of um-PEA-LUT and OT significantly improved the overall TDI score compared to OT alone (MD = 5.32, 95% CI [1.25, 9.39], p = 0.01). Significant heterogeneity in the TDI scores was identified but resolved through a sensitivity analysis, without affecting the significance of the results (MD = 6.86, 95% CI [2.82, 10.91], p = 0.0009).

**Conclusion:**

The addition of um-PEA-LUT to OT resulted in significant improvements in the overall TDI score in patients with persistent post-COVID-19 OD. This finding offers a promising therapeutic option for managing this challenging condition.

**Disclosures:**

All Authors: No reported disclosures

